# Extreme altitude induces divergent mass reduction of right and left ventricle in mountain climbers

**DOI:** 10.14814/phy2.15184

**Published:** 2022-02-11

**Authors:** Camilla Udjus, Ivar Sjaastad, Ulla Hjørnholm, Torbjørn K. Tunestveit, Pavel Hoffmann, Alexis Hinojosa, Emil K. S. Espe, Geir Christensen, Ole H. Skjønsberg, Karl‐Otto Larsen, Morten Rostrup

**Affiliations:** ^1^ Department of Pulmonary Medicine Oslo University Hospital Ullevål Oslo Norway; ^2^ Institute for Experimental Medical Research Oslo University Hospital Ullevål and University of Oslo Oslo Norway; ^3^ K.G. Jebsen Center for Cardiac Research University of Oslo Oslo Norway; ^4^ Faculty of Medicine Institute of Clinical Medicine University of Oslo Oslo Norway; ^5^ Department of Cardiology Oslo University Hospital Ullevål Oslo Norway; ^6^ Section of Cardiovascular and Renal Research Medical Division Department of Cardiology Oslo University Hospital Ullevål Oslo Norway; ^7^ University of Oslo Oslo Norway; ^8^ Section for Interventional Cardiology Division of Cardiovascular and Pulmonary Diseases Department of Cardiology Oslo University Hospital Oslo Norway; ^9^ Department of Radiology and Nuclear Medicine Oslo University Hospital Ullevål Oslo Norway; ^10^ Interventional Centre (IVS) Oslo University Hospital Rikshospitalet and University of Oslo Oslo Norway; ^11^ Department of Acute Medicine Oslo University Hospital Oslo Norway

**Keywords:** cardiac mass, cytokines, diastolic dysfunction, extreme altitude, hypobaric hypoxia

## Abstract

Mountain climbing at high altitude implies exposure to low levels of oxygen, low temperature, wind, physical and psychological stress, and nutritional insufficiencies. We examined whether right ventricular (RV) and left ventricular (LV) myocardial masses were reversibly altered by exposure to extreme altitude. Magnetic resonance imaging and echocardiography of the heart, dual x‐ray absorptiometry scan of body composition, and blood samples were obtained from ten mountain climbers before departure to Mount Everest or Dhaulagiri (baseline), 13.5 ± 1.5 days after peaking the mountain (post‐hypoxia), and six weeks and six months after expeditions exceeding 8000 meters above sea level. RV mass was unaltered after extreme altitude, in contrast to a reduction in LV mass by 11.8 ± 3.4 g post‐hypoxia (*p* = 0.001). The reduction in LV mass correlated with a reduction in skeletal muscle mass. After six weeks, LV myocardial mass was restored to baseline values. Extreme altitude induced a reduction in LV end‐diastolic volume (20.8 ± 7.7 ml, *p* = 0.011) and reduced E’, indicating diastolic dysfunction, which were restored after six weeks follow‐up. Elevated circulating interleukin‐18 after extreme altitude compared to follow‐up levels, might have contributed to reduced muscle mass and diastolic dysfunction. In conclusion, the mass of the RV, possibly exposed to elevated afterload, was not changed after extreme altitude, whereas LV mass was reduced. The reduction in LV mass correlated with reduced skeletal muscle mass, indicating a common denominator, and elevated circulating interleukin‐18 might be a mechanism for reduced muscle mass after extreme altitude.

## Graphical abstract

Climbing to extreme altitude induces divergent effects on the left and right ventricular mass, the former being reduced and the latter being unaltered. The reduction in left ventricular mass was reversed to baseline already at six weeks follow‐up. Loss of skeletal muscle mass after extreme altitude correlated with the reduction in left ventricular mass.

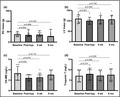

## 1 INTRODUCTION

A zest for mountain climbing has developed since expeditions to high altitude started in the middle of the 19^th^ century, and a concomitant interest in high altitude physiology emerged. High altitude expeditions expose mountaineers to a hostile environment with low temperature, wind, physical and psychological stress, nutritional insufficiencies, and hypobaric hypoxia. The severity of hypoxia influences alterations in body composition, like reduced body and skeletal muscle mass and induction of pro‐inflammatory cytokines (Dünnwald et al., 2019; Lundeberg et al., 2018; Wilkins et al., 2015), and in our study mountaineers were exposed to severe hypoxia at extreme altitude. The effect of hypobaric hypoxia on the pulmonary circulation has been an object of investigation, mainly by use of echocardiography, showing elevated pulmonary artery pressure and increased right ventricular (RV) afterload during exposure to high altitude (Boussuges et al., 2000a; Swenson, 2013; Wagner, 2010). With regard to the morphology of the left side of the heart, smaller cavity, thicker walls and decreased left ventricular (LV) mass has been reported at Mount Everest Base Camp at 5300 m (Boussuges et al., 2000a; Holloway et al., 2011, 2014; Osculati et al., 2016; Reeves et al., 1985). The smaller LV cavity is compatible with findings of reduced end‐diastolic and end‐systolic volumes, whereas in the RV, both increased and unaltered end‐diastolic volume have been found and might reflect hypoxia‐induced pulmonary hypertension, dilatation and possible RV hypertrophy (Boussuges et al., 2000a; Maufrais et al., 2019). The relation between LV and RV mass has not been examined before after extreme altitude, and whether possible changes are reversible at an early time point of six weeks follow‐up. Hypoxia can induce circulating pro‐inflammatory cytokines (Dünnwald et al., 2019; Larsen et al., 2008; Lundeberg et al., 2018; Wilkins et al., 2015), which are prone to mediate loss of muscle mass (Li et al., 2019; Petersen et al., 2007; Takenaka et al., 2014). Whether such a mechanism could exert comparable effects on both the masses of the LV and skeletal muscle is not known. In the current study, 10 mountain climbers were studied prior and subsequent to a climb to extreme altitude. We hypothesized that extreme altitude and hypobaric hypoxia induce an increase in RV mass, due to increased afterload related to pulmonary hypertension, in contrast to a reduction in LV mass. We further proposed that a reduction in LV mass would correlate with a reduction in skeletal muscle mass. Magnetic resonance imaging (MRI) and echocardiography were performed prior to ascent, 13.5 ± 1.5 days after peaking the mountain (post‐hypoxia), and six weeks and six months after expeditions to Mount Everest, (8847 m) or Dhaulagiri (8167 m). We were also able to examine possible alterations in RV and LV volume and function, and whether the changes were reversible within this time frame. Moreover, body mass composition and levels of pro‐inflammatory cytokines, which can be induced by hypoxia, were measured.

## 2 MATERIALS AND METHODS

### 2.1 Participants and setting

Seventeen volunteers were invited to the study, of which 7 were not able to fulfill the baseline or post‐hypoxia investigations due to unforeseen, not health related, incidents. Thus, our study was carried out on 10 healthy volunteers (7 male, 3 female, aged 44 ± 3 years) who participated in climbs to extreme altitude above 6000 m in the Himalayas (Table 1). The participants were informed about the procedures and gave written informed consent. Baseline examinations were performed close to the departure. The participants traveled to Kathmandu in Nepal by flight, then to expedition start points for Mt Everest (8848 m) or Dhaulagiri (8167 m) in the Himalayas. One participant did not reach the peak due to an avalanche, still he reached extreme altitude of 6500 m. The expeditions lasted 6–8 weeks, including ascent/decent and acclimatization. The participants spent two days descending from the peak of the mountain to Base Camp at 5300 m, stayed there for one day, before hiking to Kathmandu over the next two‐three days. The participants were examined in Norway 13.5 ± 1.5 days after peaking the mountain (post‐hypoxia), and at six weeks and six months follow up. The study was approved by the Norwegian Regional Committee for Medical and Health Research Ethics (#2011/587).

**TABLE 1:** Characteristics of participants of treks to extreme altitude

ID	Sex	Age	Height (cm)	Weight (kg)	Achieved height (m)	Oxygen supply (h)
1	Male	42	177	80.8	6500	0
2	Female	51	167	60.8	8167	60
3	Male	35	175	79.5	8167	20
4	Male	57	174	86.1	8848	60
5	Male	37	180	78.6	8848	60
6	Male	41	182	88.9	8848	20
7	Male	27	182	90.2	8848	60
8	Female	43	177	68.0	8848	40
9	Female	53	168	62.5	8848	60
10	Male	54	176	80.5	8848	60

### 2.2 Cardiac magnetic resonance imaging

Left and right ventricular cardiac masses and volumes were assessed using cardiac MRI, in a 1.5 T Philips clinical scanner (Philips Healthcare, Best, The Netherlands). Two and four chamber long‐axis cine images were acquired. Images with late gadolinium enhancement obtained 15 min after contrast injection (gadolinium‐DTPA 469 mg/ml, 0.15 mmol/kg; Magnevist, Schering AG, Germany) (in seven of the ten climbers) were used to analyze possible myocardial necrosis (Limalanathan et al., 2013). Two chamber short‐axis images were obtained with 8 mm thick slices covering the ventricles from the base to the apex. Thirty cardiac cycle phases were obtained. LV and RV endocardium and epicardium of the short‐axis cine images were manually contoured in a blinded manner, and reviewed by a second investigator, before the masses were calculated on a View Forum workstation (Philips Medical Systems, Best, The Netherlands). To assess LV and RV myocardial function, the cine loops were analyzed with feature tracking using Segment v3.2 R8456 (http://segment.heiberg.se) (Heiberg et al., 2010). Briefly, the LV and RV endo‐ and epicardial borders of the short‐axis cine images were manually traced before an automatic tissue tracking algorithm calculated peak LV and RV long‐axis strain and strain rates. Key imaging parameters include an in‐plane resolution of between 0.63 mm^2^ and 1.13 mm^2^, slice thickness 8 mm and a temporal resolution of 23–48 ms, depending on heart rate.

### 2.3 Echocardiography

LV and RV cardiac structure and function were assessed using two‐dimensional, M‐mode and Doppler echocardiography (Vivid 7 ultrasound scanner, GE Vingmed Ultrasound, Horten, Norway). Images were stored digitally, and analyzed (Echopac 202, GE Vingmed Ultrasound) by a single physician blinded to clinical and subject related information. Three cardiac cycles were recorded, at a minimum (Schwartz et al., 2014).

### 2.4 Body composition

Total lean body mass, fat mass, skeletal muscle mass in arms, legs and truncus, and total bone mineral content were assessed by dual x‐ray absorptiometry (DXA) using a GE Lunar Prodygi with enCore v.16 (Madison, WI). Examinations were performed by skilled radiographers. Lean mass was defined as tissue without fat and bone minerals and is referred to as skeletal muscle mass. Total bone mineral content (TBMC) is the sum of bone, presented in grams (Bonnick & Lewis, 2013).

### 2.5 Blood samples

Ten milliliters of venous blood from the participants were collected in vacutainer tubes. Serum samples were allowed to clot for 60 min at room temperature, before separated by 15 min centrifugation at 3500 rpm. Plasma samples were collected in ethylenediaminetetraacetic acid (EDTA) tubes (BD, Plymouth, UK), stored on crushed ice immediately after sampling and centrifuged at 3000 *g* for 20 min at 4°C. All samples were aliquoted and stored at −80°C, thawed only once, and analyzed immediately. Hematocrit and hemoglobin were measured in EDTA whole blood, and cholesterol, creatinine kinase (CK), CK‐MB, troponin T (TnT) and pro‐brain natriuretic peptide (proBNP) were measured in serum. By commercially available ELISA‐kits (Human interleukin (IL)‐18/IL‐1F4 ELISA #7620, Human IL‐6 Quantikine ELISA #D6050, and Human IFN‐ɣ Quantikine ELISA #DIF50C, all from R&D systems, Minneapolis, MN), protein levels of IL‐18, IL‐6, and IFN‐ɣ were measured in serum. Each sample was assayed in duplicate on 96‐well microplates according to the manufacturer's instructions, and detection carried out with a Hidex Microplate Reader (Hidex, Turku, Finland).

### 2.6 Statistical analysis

Data are presented as mean ± standard deviation (SD) if normally distributed, otherwise as median (range). Normality analyses were performed by GraphPad Prism (San Diego, CA). Differences between values at different time points were tested using repeated measurement ANOVA, followed by Dunnett's post hoc test for parametric statistics, or by an overall Friedman test, followed by Dunn's test post hoc for non‐parametric data. *p*‐value < 0.05 was considered statistically significant.

## 3 RESULTS

### 3.1 Unchanged RV mass and reduced LV mass after extreme altitude

**FIGURE 1:**
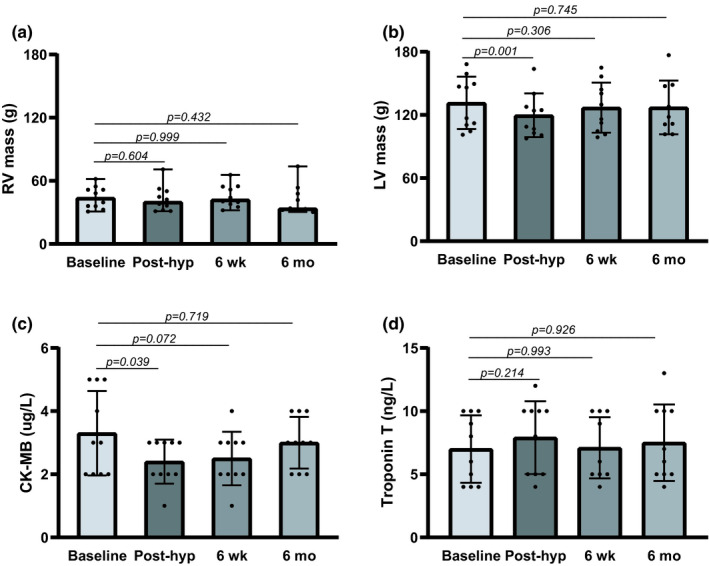
Cardiac masses and biomarkers in mountain climbers before (baseline) and after extreme altitude. Post‐hyp, post‐hypoxia; 6 wk, six weeks; 6 mo, six months; RV, right ventricular; LV, left ventricular; CK‐MB, creatinine kinase‐MB. Overall test *p*‐values; (a) *p* = 0.228, (b) *p* = 0.004, (c) *p* = 0.047, (d) *p* = 0.567

Exposure to extreme altitude and hypobaric hypoxia did not induce significant changes in RV mass (Figure 1a). In the LV, however, an 11.8 g (9.0%, *p* = 0.001) reduction in myocardial mass was observed after extreme altitude (Figure 1b). At six weeks and six months follow‐up, LV mass was restored to baseline values (Figure 1b). CK‐MB values were reduced by 27.3% post‐hypoxia (*p* = 0.039), corresponding with a reduction in LV myocardial mass (Figure 1c). There was no late gadolinium enhancement present after extreme altitude, indicating no signs of myocardial necrosis. This finding corresponds with no significant changes in Troponin T, a marker of cardiac muscle damage, post‐hypoxia (Figure 1d).

### 3.2 Body composition after extreme altitude

**FIGURE 2:**
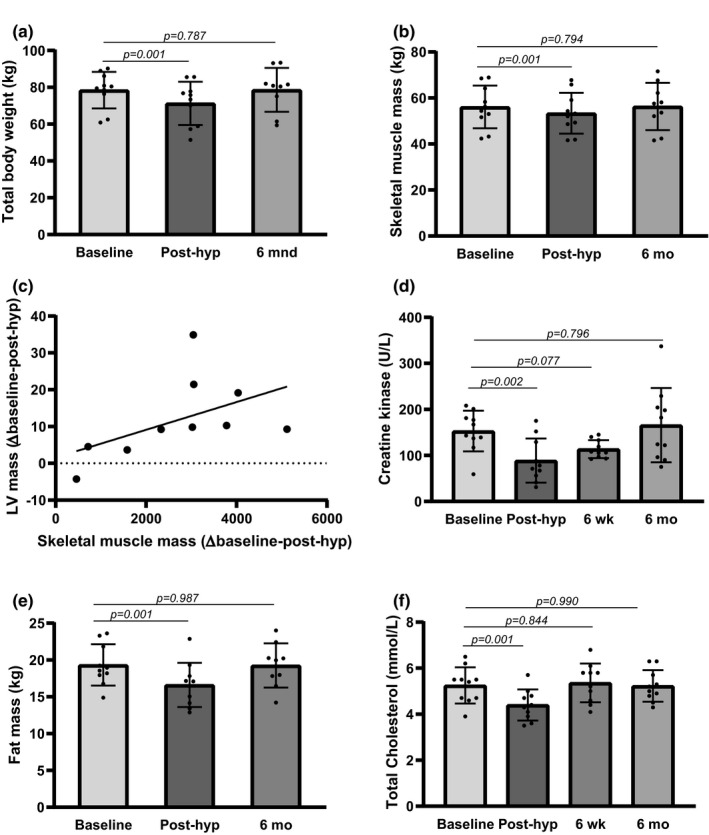
Body composition in mountain climbers before (baseline) and after extreme altitude. Post‐hyp, post‐hypoxia; 6 wk, six weeks; 6 mo, six months; LV, left ventricular. Overall test *p*‐values; (a) *p* = 0.001, (b) *p* = 0.001, (d) *p* = 0.001, (e) *p* = 0.001, (f) *p* = 0.001

Body weight was reduced by 7.2 kg (9.2%, *p* = 0.001) post‐hypoxia (Figure 2a), with a 4.8% reduction in skeletal muscle mass (*p* = 0.001) (Figure 2b). A correlation in differences between baseline and post‐hypoxia values for skeletal muscle mass and LV mass was found (*r* = 0.697, *p* = 0.013) (Figure 2c), suggesting a common denominator. The reduction in skeletal muscle mass was found in both arms (11.1%), legs (6.5%), and truncus (1.5%). The reduction in kilograms was most profound in the legs (1.2 kg). For truncus alone, the muscle mass reduction was not significant after extreme altitude. After 6 months, skeletal muscle mass showed no significant change from baseline values (Figure 2b). In the blood, extreme altitude induced a 41.9% (*p* = 0.002) reduction in CK from baseline, which may reflect loss of muscle after hypoxic exposure (Figure 2d). A reduction of 2.7 kg (14.0%, *p* = 0.001) in fat mass was measured after hypobaric hypoxia (Figure 2e), and a decrease in total cholesterol was found (16.2%, *p* = 0.001) in blood post‐hypoxia (Figure 2f). The body weight and fat mass were not altered from baseline at six months follow‐up, showing reversibility after extreme altitude (Figure 2a and e). Overall test for TMBC showed *p* = 0.016. At baseline TMBC values were 3058 ± 485.8 g which decreased to 3020 ± 472.7 g (*p* = 0.037) after extreme altitude. In contrast to fat mass and skeletal muscle mass which were reversible after six months follow‐up, TBMC was still decreased by 0.5% (*p* = 0.019) at six months follow‐up to values of 3027 ± 496.7 g, showing slower recovery of bone mineral content. Taken together, extreme altitude induced reversible reductions in total body mass, skeletal muscle mass and fat mass. TBMC was still decreased at six months follow‐up.

### 3.3 RV afterload and LV diastolic dysfunction after extreme altitude

**FIGURE 3:**
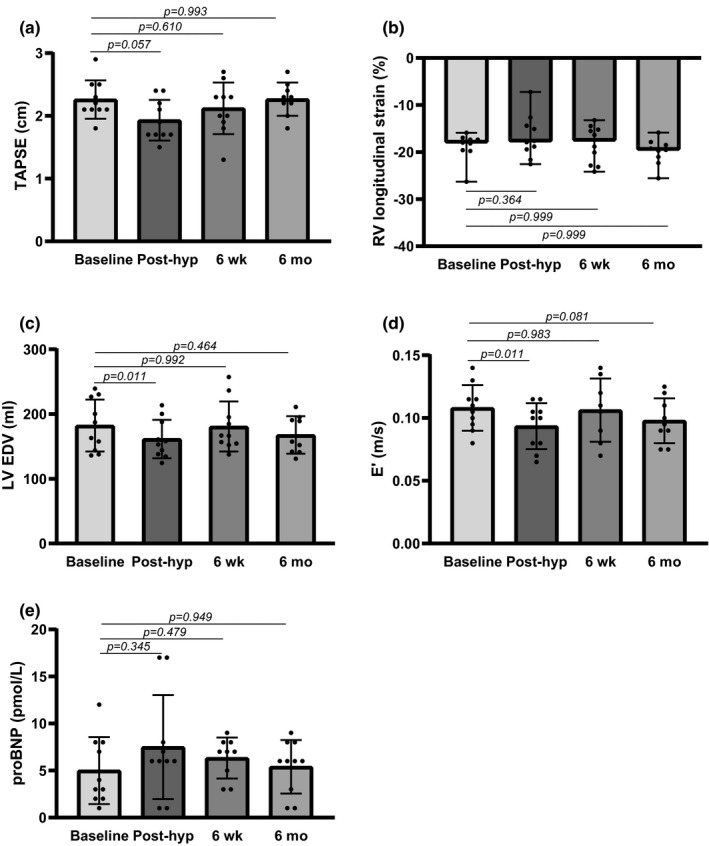
Right ventricular afterload and left ventricular diastolic function in mountain climbers before (baseline) and after extreme altitude. Post‐hyp, post‐hypoxia; 6 wk, six weeks; 6 mo, six months; TAPSE, tricuspid annular plane systolic excursion; RV, right ventricular; LV EDV, left ventricular end diastolic volume; E’, peak velocity of early diastolic mitral annular motion; proBNP, pro‐brain natriuretic peptide. Overall test *p*‐values; (a) *p* = 0.056, (b) *p* = 0.176, (c) *p* = 0.015, (d) *p* = 0.012, (e) *p* = 0.237

RV hypertrophy was not found, and no significant changes in tricuspid annular plane systolic excursion (TAPSE) (14.6%, *p* = 0.056) (Figure 3a) were found post‐hypoxia by echocardiography. MRI analysis showed no significantly altered RV longitudinal strain post‐hypoxia (Figure 3b). RV EF, RV EDV and RV ESV were not significantly changed after extreme altitude, indicating preserved RV systolic function without dilatation after extreme altitude (Table 2). Moreover, velocity of blood flow across the pulmonary valve, PV Vmax was unaltered post‐hypoxia (Table 3), and no tricuspid regurgitation was detected (Table 3). In the LV, EDV decreased with 11.4% (*p* = 0.011) post‐hypoxia (Figure 3c). Peak LV myocardial relaxation, estimated by peak velocity of early diastolic mitral annular motion (E′), was reduced by 13.4% (*p* = 0.011) post‐hypoxia (Figure 3d), indicating diastolic dysfunction. The mitral inflow velocity (E)/E′ ratio was not significantly changed post‐hypoxia (Table 3). Preserved LV systolic function after extreme altitude was shown by unchanged EF, CO, SV, and ESV (Table 2). ProBNP was not significantly changed post‐hypoxia (*p* = 0.345) (Figure 3e). Taken together, examination of cardiac function showed no significant changes in measurements of RV afterload, whereas a LV diastolic dysfunction was detectable after extreme altitude.

**TABLE 2:** Cardiac volumes and systolic function after extreme altitude assessed by magnetic resonance imaging

	Baseline (*n* = 10)	Post‐hyp (*n* = 10)	6 wk (*n* = 10)	6 mo (*n* = 9)	Overall test *p*‐value	Post hoc test *p*‐value		
						Baseline vs. post‐hyp	Baseline vs. 6 wk	Baseline vs. 6 mo
Right ventricle								
End diastolic volume (ml)	212 ± 59	203 ± 63	218 ± 55	191 ± 47	0.052	0.364	0.631	0.310
End systolic volume (ml)	100 ± 49	98 ± 41	102 ± 36	83 ± 31	0.326	0.986	0.954	0.365
Stroke volume (ml)	112 ± 15	105 ± 24	116 ± 22	105 ± 17	0.196	0.385	0.811	0.802
Ejection fraction (%)	55 ± 11	53 ± 5.4	54 ± 5.9	57 ± 5.4	0.419	0.627	0.934	0.844
Cardiac output (L/min)	6.9 ± 1.2	6.4 ± 1.6	6.8 ± 1.2	6.3 ± 1.2	0.594	0.584	0.992	0.575
Left ventricle								
End systolic volume (ml)	72 ± 25	65 ± 17	72 ± 18	61 ± 18	0.102	0.210	0.999	0.148
Stroke volume (ml)	110 ± 20	96 ± 14	109 ± 27	107 ± 15	0.054	0.044	0.989	0.994
Ejection fraction (%)	61 ± 8	60 ± 3	60 ± 6	64 ± 6	0.246	0.910	0.899	0.050
Cardiac output (L/min)	6.8 ± 1.3	5.9 ± 1.1	6.4 ± 1.5	6.3 ± 0.9	0.284	0.140	0.747	0.633

Note

Data are presented as mean ± SD.

Abbreviations: 6 wk, six weeks; 6 mo, six months.

**TABLE 3:** Cardiac function after extreme altitude assessed by echocardiography

	Baseline (*n* = 10)	Post‐hyp (*n* = 10)	6 wk (*n* = 10)	6 mo (*n* = 9)	Overall test *p*‐value	Post hoc test *p*‐value		
						Baseline vs. post‐hyp	Baseline vs. 6 wk	Baseline vs. 6 mo
Right ventricle								
PV Vmax (m/s)	0.9 (0.6, 1.2)	0.9 (0.7, 1.3)	0.9 (0.7, 1.2)	0.8 (0.6, 1.2)	0.173	0.999	0.999	0.281
TR Vmax (m/s)	1.5 ± 0.6	1.5 ± 0.5	1.6 ± 0.4	1.6 ± 0.5	0.614	0.963	0.434	0.835
TR max PG	9.9 ± 6.9	10.2 ± 7.2	10.8 ± 5.6	10.8 ± 6.2	0.738	0.992	0.585	0.969
Right atrial area (cm^2^)	17.0 ± 3.2	17.3 ± 3.2	17.9 ± 2.5	18.3 ± 3.6	0.673	0.989	0.741	0.564
Left ventricle								
Left atrial diameter (cm)	3.7 ± 0.4	3.4 ± 0.5	3.5 ± 0.4	3.6 ± 0.5	0.076	0.038	0.587	0.937
Left atrial area (cm^2^)	17.2 ± 3.3	16.2 ± 2.5	16.9 ± 1.8	18.1 ± 2.6	0.183	0.455	0.957	0.618
MV E velocity (m/s)	0.6 ± 0.1	0.6 ± 0.1	0.7 ± 0.2	0.6 ± 0.1	0.020	0.070	0.669	0.999
MV A velocity (m/s)	0.5 ± 0.1	0.5 ± 0.1	0.6 ± 0.1	0.5 ± 0.1	0.012	0.273	0.133	0.968
MV E/A ratio	1.3 ± 0.2	1.2 ± 0.3	1.2 ± 0.3	1.2 ± 0.3	0.889	0.842	0.828	0.948
MV Dec T (ms)	273.1 ± 40.6	271.3 ± 64.8	265.3 ± 48.6	263.4 ± 55.5	0.883	0.994	0.917	0.910
MV Dec slope (m/s^2^)	2.5 ± 0.7	2.2 ± 0.8	2.7 ± 1.0	2.4 ± 0.7	0.369	0.672	0.724	0.910
MV E velocity/E′	5.9 ± 1.2	6.1 ± 1.5	6.3 ± 1.7	6.2 ± 0.7	0.182	0.865	0.338	0.114
Long. strain 4 chamber	17.7 ± 1.7	17.6 ± 2.3	17.8 ± 1.8	18.2 ± 3.5	0.889	0.996	0.998	0.885
Long. strain 2 chamber	19.6 ± 2.0	20.0 ± 2.3	20.3 ± 1.8	19.2 ± 2.2	0.596	0.906	0.689	0.963
Circumferential strain MV	18.7 ± 3.8	18.7 ± 4.1	18.4 ± 4.3	20.0 ± 3.2	0.586	0.999	0.993	0.589
MAPSE lateral (cm)	1.5 ± 0.2	1.5 ± 0.2	1.4 ± 0.3	1.5 ± 0.2	0.688	0.735	0.508	0.903
MAPSE medial (cm)	1.3 ± 0.3	1.3 ± 0.2	1.4 ± 0.3	1.3 ± 0.1	0.237	0.967	0.268	0.922

Note

Data are presented as mean ± SD or median (range).

Abbreviations: 6 mo, six months; PV, pulmonary valve; 6 wk, six weeks; MAPSE, mitral annular plane systolic excursion; MV, mitral valve; PG, pressure gradient; TR, tricuspid regurgitation.

### 3.4 Pro‐inflammatory cytokines after extreme altitude

Circulating levels of IL‐18 were elevated post‐hypoxia compared to six months follow‐up (Table 4). Levels of the pro‐inflammatory cytokines IFN‐ɣ and IL‐6, both able to influence muscle mass and cardiac function, were not changed post‐hypoxia (Table 4). Hematocrit and hemoglobin were increased after extreme altitude (Table 4).

**TABLE 4:** Pro‐inflammatory cytokines, hematocrit, and hemoglobin after extreme altitude

	Baseline (*n* = 10)	Post‐hyp (*n* = 10)	6 wk (*n* = 10)	6 mo (*n* = 10)	Overall test *p*‐value	Post hoc test *p*‐value		
						Baseline vs. post‐hyp	Baseline vs. 6 wk	Baseline vs. 6 mo
IL‐18 (pg/ml)	209.4 (94.5, 342.3)	278.2 (54.0, 862.8)^*^	164.0 (34.3, 320.0)	173.0 (41.0, 246.0)	0.006	0.865	0.999	0.267
IFN‐ɣ (pg/ml)	15.4 (14.5, 17.0)	17.4 (14.7, 19.4)	15.3 (14.3, 21.1)	15.7 (14.4, 37.2)	0.208	0.301	0.999	0.999
IL‐6 (pg/ml)	3.2 (2.5, 10.9)	4.4 (2.8, 22.1)	3.3 (2.5, 11.0)	3.4 (1.7, 36.5)	0.269	0.204	0.999	0.999
Hematocrit	0.43 ± 0.02	0.48 ± 0.04	0.44 ± 0.03	0.43 ± 0.03	0.001	0.001	0.648	0.995
Hemoglobin (g/dl)	14.6 ± 1.00	16.1 ± 1.02	14.6 ± 1.08	14.6 ± 1.25	0.001	0.001	0.999	0.997

Note

Data are presented as mean ± SD or median (range).

^*^ *p* = 0.003, post‐hypoxia vs. 6 mo.

## 4 DISCUSSION

In this study we have shown that exposure to extreme altitude and hypobaric hypoxia leads to a reduction in LV mass and end‐diastolic volume, while RV mass and volumes were not significantly impacted. The reduction in LV mass correlated with a more modest decrease in skeletal muscle mass post‐hypoxia. The changes in LV were reversed already after six weeks at sea level.

This is, to our knowledge, the first study comparing RV and LV mass and function by MRI and echocardiography after extreme altitude and severe hypobaric hypoxia. Arterial oxygen saturation drops rapidly to approximately 70% at 6000 m, and further ascendance leads to more profound desaturation, as reported by Grocott et al of a mean arterial oxygen value of 24.6 mmHg (3.28 kPa) at 8400 m on descent from the summit of Mt Everest (Grocott et al., 2010). Houston and co‐workers simulated ascent of Mt Everest in an altitude chamber and assessed the development of hypoxia‐induced pulmonary hypertension by right heart catheterization (Groves et al., 1985). Pulmonary hypertension occurred from a simulated altitude of 3000 m (Groves et al., 1985). In the field, pulmonary hypertension has been shown from an altitude of 2500 m (Bartsch & Swenson, 2013). With regard to RV systolic performance, the results are more diverging. In a field study at high altitude (5050 m) impaired RV systolic performance, shown by reduced RV strain, was observed (Stembridge et al., 1985). This is in contrast to a study after one hour in normobaric hypoxia (FiO_2_ 12.5%) in which an enhanced RV systolic function, evident by increased TAPSE, was demonstrated (Kjaergaard et al., 2007). In our study, no significant changes in RV function, including longitudinal strain, were detectable two weeks after hypobaric hypoxia. It is well established that prolonged pulmonary hypertension causes RV hypertrophy due to elevated afterload (Swenson, 2013). By using magnetic resonance imaging, the gold standard for examination of cardiac mass and volumes, we found no significant changes in RV mass after exposure to extreme altitude. Thus, an expected RV hypertrophy post‐hypoxia did not materialize. The unaltered RV mass could indicate that other mechanisms, like those inducing reduced LV mass, may counteract the hypertrophic stimulus of elevated afterload.

We report a 9% reduction in LV mass after extreme altitude and a more modest decrease of 4.8% in skeletal muscle mass. Interestingly, a significant correlation between the reduction in LV mass and skeletal muscle mass was found post‐hypoxia. Holloway and co‐workers also documented reduced LV mass by MRI, whereas skeletal muscle mass was unaltered after their expedition to Mt Everest Base Camp (5300 m) (Holloway et al., 2011). They put forward that the hypoxia‐induced reduction in LV mass can be an autophagic process, analogous to wasting of skeletal muscle mass or reduced protein synthesis, mechanisms which might explain our findings of reduced LV and skeletal muscle mass after extreme altitude (Holloway et al., 2011, 2014; Howald & Hoppeler, 2003). Other possible mechanisms for reduction in LV and skeletal muscle mass are muscle deconditioning or detraining related to less activity, like physical inactivity during acclimatization and lower workloads at extreme altitude (Ferrando et al., 1996; Pedlar et al., 1985; Perhonen et al., 1985). There was no late gadolinium enhancement present at any time point, indicating that there was no myocardial necrosis after extreme altitude. Hypoxia, affecting both the LV and RV, can induce apoptosis of cardiomyocytes via endoplasmatic reticulum stress (Luo et al., 2015). High altitude and hypobaric hypoxia leading to reduced LV mass may have similar effects on the RV. Hence, processes of cardiac muscle wasting, may occur in LV and RV, whereas in the RV hypertrophic stimuli due to possible elevated afterload may result in our finding of unaltered RV mass post‐hypoxia.

Skeletal muscle wasting seems to be related to the extent of altitude and ensues predominantly at altitudes above 5000 m (D'Hulst & Deldicque, 1985). In fact, the severity of hypoxia, which increases with altitude, correlates negatively with percentage decrease in muscle fiber area, suggesting that hypoxia is important in the reduction of muscle mass after a hypoxic sojourn (D'Hulst & Deldicque, 1985). Thus, the discrepancy in loss of skeletal muscle mass between studies at Mt Everest Base Camp (Holloway et al., 2011, 2014) and our study at extreme altitude can be due to different levels of altitude and hypoxic dose. Interestingly, the few studies which have investigated skeletal muscle mass and LV mass after high altitude, show no reduction in skeletal muscle mass post‐hypoxia (Holloway et al., 2011, 2014), whereas LV mass was reduced, indicating a LV vulnerability at both high and extreme altitudes. In addition to hypoxia, the low barometric pressure at Mt Everest summit (253 mmHg or 1/3 of barometric pressure at sea level) can reduce intrathoracic pressure due to the decreased gravitational compression. Effects of reduced gravity and loading on the LV have been shown during spaceflight and horizontal bed rest for six weeks, where a gravity of nearly zero or horizontal position in bed induced a LV atrophy of 8% in spite of continuous oxygen supply in the spacecraft (Henry et al., 1974; Perhonen et al., 1985). Hence, lower barometric pressure, occurring at Mt Everest summit, may have contributed to the reduced LV mass observed in the current study, in synergy with hypoxia. Other processes in the body, such as bone mineralization, are also hampered in low atmospheric pressure (Penttinen et al., 1972; Tanaka et al., 1992). Thus, reduced atmospheric pressure can, at least partly in our study, explain both reduced total bone mineral content and LV mass after extreme altitude.

LV end‐diastolic volume was reduced by 11% post‐hypoxia, which has been related to the well‐known reduction in plasma volume at high altitude, that also contributes to the observed increase in hemoglobin and hematocrit values, together with hypoxia‐induced erythrocytosis (Pugh, 1964). Reduction in LV EDV has been observed by others (Fowles & Hultgren, 1983; Maufrais et al., 2019; Osculati et al., 2016; Stembridge et al., 1985), as well, and it was interesting to observe that this reduction was still evident nearly two weeks after descent. The decrease in LV volume and mass were reversed to baseline values at six weeks follow‐up, showing earlier restoration of LV mass than previously shown (Holloway et al., 2011). In our study, reduced E’ indicates impaired left heart myocardial relaxation post‐hypoxia. Impairment of the early phase of diastole has been shown at high altitude and during experimental hypobaric hypoxia (Huez et al., 2005; Kjaergaard et al., 2006; Osculati et al., 2016; Reeves et al., 1985). Reduction in LV preload post‐hypoxia, evident by reduced LV EDV, may decrease LV early filling (Boussuges et al., 2000b). Another possible mechanism for impaired LV relaxation is diastolic chamber stiffness due to reduced energy supply in hypoxia (Gomez & Mink, 1992). In the cardiomyocytes, the rate of relaxation is primarily regulated by removal of cytosolic Ca^2+^ by the activity of the sarcoendoplasmic reticulum Ca^2+^ ATPase pump (SERCA), an ATP consuming process. Reduced cardiac phosphocreatine/ATP ratio was found after expedition to high altitude and in experimental hypoxia, which can decrease energy available for the SERCA pump and lead to diastolic dysfunction (Holloway et al., 2011; Portman et al., 1996). The SERCA pump activity is regulated by phosphorylation of phospholamban, and in animal studies we have previously shown that hypoxia‐induced IL‐18, leading to reduced phosphorylation of phospholamban, can inhibit SERCA activity and impair myocardial relaxation (Larsen et al., 2006, 2008). In this study, circulating IL‐18 was elevated post‐hypoxia compared to follow‐up, and IL‐18‐mediated development of diastolic dysfunction after hypobaric hypoxia is a possibility. Interestingly, increased circulating levels of IL‐18 have been found in patients with decreased skeletal muscle mass, including sarcopenia, heart failure, and chronic obstructive pulmonary disease (COPD) (Imaoka et al., 2008; Li et al., 2019; Petersen et al., 2007; Seta et al., 2000). The skeletal muscle alterations in COPD, heart failure, and deconditioning/detraining are similar, including atrophy, apoptosis, and altered fiber type distribution to a faster phenotype (Rehn et al., 2012). Experimentally, overproduction of IL‐18 in transgenic mice induced loss of muscle weight (Takenaka et al., 2014), indicating that IL‐18 might be a contributing mechanism for muscle loss in our study, as well.

A limitation of our study is the modest sample size which may conceal possible differences between values obtained at baseline and at the various time points. Strengths of the study are that both cardiac MRI and echocardiography are performed at four time points, before and after climbs to extreme altitude. Simultaneous examination of body composition and blood samples enabled comparison of alterations in the heart with changes in other organs as well as levels of circulating cytokines.

In conclusion, climbing to extreme altitude resulted in diverging effects on RV and LV mass, being unaltered and reduced, respectively. The unaltered RV mass indicates counteracting stimuli on the RV myocardium; hypertrophy related to increased pulmonary arterial pressure and afterload, and myocardial wasting, as observed in the LV, induced by hypobaric hypoxia. Reduction in LV mass correlated with loss of skeletal muscle mass, both being reversed to baseline values after 6 weeks at sea level. Elevated levels of IL‐18 might be studied further as a common contributing mechanism for cardiac remodelling and loss of muscle mass at extreme altitude (Lankford & Swenson, 2014).

## CONFLICT OF INTEREST

No conflicts of interest, financial or otherwise, are declared by the authors.

## AUTHOR CONTRIBUTION

C.U. analyzed data and drafted manuscript; I.S. performed echocardiography and drafted manuscript; U.H. designed study and coordinated the examinations of the study participants; T.K.T. analyzed data; P.H. contributed to MRI examinations and interpreted results; A.H. contributed to DXA scan examination; E.K.S.E. analyzed data; G.C., O.H.S., and K.O.L. drafted manuscript, and M.R. conceived and designed research and drafted manuscript.
